# A Mobile Phone App Featuring Cue Exposure Therapy As Aftercare for Alcohol Use Disorders: An Investigator-Blinded Randomized Controlled Trial

**DOI:** 10.2196/13793

**Published:** 2019-08-16

**Authors:** Angelina Isabella Mellentin, Bent Nielsen, Anette Søgaard Nielsen, Fei Yu, Anna Mejldal, Dorthe Grüner Nielsen, Elsebeth Stenager

**Affiliations:** 1 Unit for Clinical Alcohol Research, Unit for Psychiatric Research Department of Clinical Research University of Southern Denmark Odense Center Denmark; 2 Brain Research-Inter-Disciplinary Guided Excellence Department of Clinical Research University of Southern Denmark Odense Center Denmark; 3 Unit for Psychiatric Research Institute of Regional Health Services Research University of Southern Denmark Aabenraa Denmark; 4 Technology Entrepreneurship and Innovation section Mads Clausen Institute University of Southern Denmark Sønderborg Denmark

**Keywords:** alcohol use disorder, aftercare, cue exposure therapy, cognitive behavior therapy, randomized controlled trial, mobile phone app

## Abstract

**Background:**

Cue exposure therapy (CET) is a psychological approach developed to prepare individuals with alcohol use disorder (AUD) for confronting alcohol and associated stimuli in real life. CET has shown promise when treating AUD in group sessions, but it is unknown whether progressing from group sessions to using a mobile phone app is an effective delivery pathway.

**Objective:**

The objectives of this study were to investigate (1) whether CET as aftercare would increase the effectiveness of primary treatment with cognitive behavior therapy, and (2) whether CET delivered through a mobile phone app would be similarly effective to CET via group sessions.

**Methods:**

A total of 164 individuals with AUD were randomized to one of three groups: CET as group aftercare (CET group), CET as fully automated mobile phone app aftercare (CET app), or aftercare as usual. Study outcomes were assessed face-to-face at preaftercare, postaftercare, and again at 6 months after aftercare treatment. Generalized mixed models were used to compare the trajectories of the groups over time on drinking, cravings, and use of urge-specific coping skills (USCS).

**Results:**

In all, 153 of 164 individuals (93%) completed assessments both at posttreatment and 6-month follow-up assessments. No differences in the trajectories of predicted means were found between the experimental groups (CET group and app) compared with aftercare as usual on drinking and craving outcomes over time. Both CET group (predicted mean difference 5.99, SE 2.59, *z*=2.31, *P*=.02) and the CET app (predicted mean difference 4.90, SE 2.26, *z*=2.31, *P*=.02) showed increased use of USCS compared to aftercare as usual at posttreatment, but this effect was reduced at the 6-month follow-up. No differences were detected between the two experimental CET groups on any outcomes.

**Conclusions:**

CET with USCS delivered as aftercare either via group sessions or a mobile phone app did not increase the effectiveness of primary treatment. This suggests that CET with USCS may not be an effective psychological approach for the aftercare of individuals treated for AUD.

**Trial Registration:**

ClinicalTrials.gov NCT02298751; https://clinicaltrials.gov/ct2/show/NCT02298751

## Introduction

### Background

In Western societies, individuals with an alcohol use disorder (AUD) are constantly exposed to alcohol and associated cues in their everyday life. Even when successfully treated with evidence-based psychological approaches, such as cognitive behavioral therapy, this pervasive exposure can induce cue-controlled cravings and lead to relapses, with devastating personal, familial, and socioeconomic consequences and increased burden on national health care resources.

Cue exposure therapy (CET) is a behavioristic, psychological approach to treating AUD that aims to reduce cue-induced cravings by repeatedly exposing individuals with AUD to relevant alcohol cues and hindering their habitual drinking response [1-5]. AUD individuals can thus reduce their cue reactivity and be better prepared to navigate in society.

Although CET seems to target one of the main challenges for relapse, a recent meta-analysis of controlled trials of CET for the treatment of AUD found no effect to small overall additional effects when CET was compared with other active control conditions. However, analysis of a priori defined trial covariates indicated that the type of CET and active comparison might be crucial for its effectiveness. CET combined with urge-specific coping skills (USCS) proved to be a better option for treating AUD than conventional CET [6]. During CET with USCS, individuals are initially taught coping skills and are then exposed to their preferred alcohol to generate cue-induced cravings. When the cravings peak in intensity during the exposure, the individual is actively encouraged to apply a USCS to reduce the cravings to a manageable level. This is in contrast to more conventional CET approaches in which cravings are expected to decrease without the use of USCS [7,8]. The meta-analysis also indicated that CET may prove more effective when compared with other active control conditions than cognitive behavioral therapy [6]. This is expected because cognitive behavioral therapy is one of the most effective evidence-based psychological interventions for AUD [9-12]. Furthermore, cognitive behavioral therapy has much in common with CET, especially when combined with USCS (eg, thinking about negative or positive consequences of alcohol consumption), and often integrates CET when treating other psychiatric disorders (eg, [13-15]). Currently, cognitive behavioral therapy and CET are segregated when targeting AUD and other substance use disorders, in which the emphasized difference is the in vivo exposure element featured in CET. No study has compared CET with USCS to cognitive behavioral therapy, and it is therefore not possible to disentangle the effects of type of CET from the type of comparison treatment. An important research question is whether CET combined with USCS increases the effectiveness of cognitive behavioral therapy as patients can practice coping skills while they are exposed to alcohol cues in vivo and thus experiencing cue-induced cravings.

When offering evidence-based primary and add-on aftercare AUD treatments, such as CET with USCS, the duration of the treatment is often shortened and may even be performed in group settings rather than individual sessions [10]. Due to the heavy burden on health care resources, this in itself may be an advantage of implementing evidence-based treatment strategies. Moreover, we may be experiencing another paradigm change in treatment delivery pathways, progressing from individual and group sessions to eHealth interventions through mobile devices [16-20], which could also lower the costs of treatment. Group therapies are effective for many psychological approaches [21], but little is known about the effectiveness of psychological interventions delivered through eHealth interventions, such as computers, tablets, and mobile phones [17,21-24]—and especially when delivered through a mobile phone app [23,25-27]. Preliminary evidence indicates that CET is equally effective in group settings as in individual sessions [28-30], but we do not know if a mobile phone app featuring CET with USCS would be as effective as CET delivered in group sessions.

### Objectives

The objectives of this study are two-fold: (1) to investigate whether CET with USCS (based on a published treatment manual) delivered as aftercare would increase the effectiveness of cognitive behavioral therapy compared with aftercare as usual and (2) to investigate whether CET with USCS delivered through a mobile phone app would be noninferior to CET with USCS via group sessions. In light of available evidence, it was hypothesized that the experimental CET aftercare groups would achieve superior outcomes (such as alcohol consumption, urges or cravings, and coping skills) compared with active controls treated with aftercare as usual. It was an explorative research question whether a CET mobile phone app was noninferior to the group therapy.

## Methods

The trial was registered in ClinicalTrials.gov (ID: NCT02298751) on November 6, 2014, and was conducted based on the CONSORT-EHEALTH (Consolidated Standards of Reporting Trials of Electronic and Mobile HEalth Applications and onLine TeleHealth) statement [31,32]. The CONSORT flow diagram is presented in Multimedia Appendix 1. The study methods are presented in more detail elsewhere [33,34].

### Study Design and Setting

The CET aftercare study was conducted as a single-site, investigator-blinded, parallel randomized controlled trial (RCT) in an outpatient alcohol treatment clinic in Funen, Denmark. Most individuals with AUD are offered outpatient treatment when seeking treatment in Denmark. The public outpatient treatment is paid via taxes and is open for self-referral, and patients can remain anonymous during treatment [35]. Only alcohol problems are treated at the outpatient clinic; individuals with mainly illegal drug abuse are treated elsewhere. Individuals with alcohol and/or illegal drug abuse in combination with major psychiatric disorders (schizophrenia, bipolar disorders) are also treated elsewhere.

### Primary Treatment at the Outpatient Alcohol Clinic

The primary treatment lasts 3 months and consists of both pharmacological and psychological treatment. At treatment entry, the individual is offered detoxification, if this is needed, and other pharmacological treatment (eg, disulfiram, acamprosate, naltrexone) as appropriate. The psychological treatment consists of cognitive behavioral therapy provided during 1-hour individual or group sessions and usually consists of eight sessions. The treatment course is planned together with the individual, and the therapy typically incorporates psychoeducation, functional analysis of drinking situations, development of coping strategies (eg, waiting out until the urges pass, thinking about the negative consequences of drinking, thinking about positive consequences of sobriety, and intake of alternative food and beverage), problem solving, and homework between sessions. The psychological treatments are delivered by therapists who are nurses and social workers educated within the treatment range. Supervision is frequent, and psychiatrists regularly monitor the treatment course [36].

### Recruitment

Study participants were recruited from June 1, 2015, to June 1, 2017. Shortly before concluding the standard 3-month primary treatment at the outpatient clinic, the individuals were briefly informed about the aftercare project. The information was given in the second-last session of the treatment, during which the patients were given written information about the aftercare study and were asked by the therapist if they would be willing to meet with a research assistant immediately after the end of treatment to learn more about the aftercare project. Willing individuals then received additional oral and written project information from the research assistant. After informed consent was obtained, the preaftercare interview was carried out, and individuals fulfilling the eligibility criteria were randomized to one of the three aftercare treatment groups described subsequently.

### Eligibility Criteria

To be eligible for inclusion in the study, individuals had to provide informed written consent, be aged between 18 and 80 years, and have completed primary treatment. Individuals who did not speak Danish, had an acute psychotic disorder, severe cognitive impairment, or terminal somatic illness were not eligible to participate.

### Randomization

Randomization occurred using computerized urn randomization. To ensure adequate allocation concealment, the random allocation sequence was generated by a statistician so that the research team was not involved in generating the sequence. In contrast to simple or block randomization, urn randomization is dynamic, and the probability of treatment assignment changes depending on the degree of treatment imbalance throughout the trial. Increased probability of randomization to the groups with the least number of participants increases systematically. This ensures allocation balance throughout the study and random allocation of covariates.

### Experimental and Control Aftercare Groups

Individuals who fulfilled the eligibility criteria were randomized to one of three aftercare treatment groups: (1) CET as group aftercare (CET group), (2) CET as a mobile phone app aftercare (CET app), or (3) aftercare as usual.

#### Cue Exposure Therapy Aftercare Delivered as Group Therapy

The CET aftercare in groups was conducted according to Monty and coworkers’ treatment manual for CET with USCS [28], which emphasizes the importance of individuals being confronted with alcohol to reduce cue-induced cravings. During each CET session, the individual is introduced or reintroduced to effective USCS and afterward required to practice the learned strategies while exposed to alcohol in vivo. The recommended coping strategies are (1) waiting it out, (2) thinking about the negative consequences of drinking, (3) thinking about the positive consequences of sobriety, and (4) alternative food and beverage intake. Participants were required to turn up for therapy every other week for 8 weeks (four sessions of 120 minutes each, with a maximum of eight patients in each group). Similar to cognitive behavioral therapy, the aftercare treatment was delivered by therapists (nurses and social workers trained for the purpose), and frequent supervision was conducted by a psychiatrist specialized in psychotherapy throughout the treatment phase of the study. Fidelity to the aftercare treatment manual was ensured by assessing and analyzing a 10% random sample of audio-recorded group treatment sessions.

#### Cue Exposure Therapy Aftercare Via a Mobile Phone App

**Figure 1:**
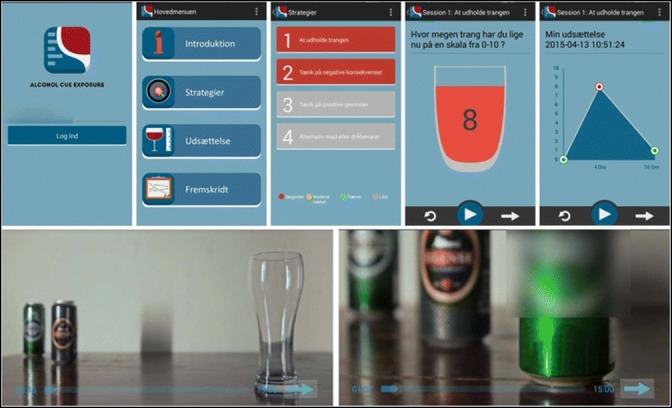
Illustration of the mobile phone app for cue exposure therapy.

Based on the same treatment manual as the group CET approach, the CET aftercare intervention was transformed into a fully automated mobile phone app (see Figure 1).

The app was individually adaptive in terms of the featured coping strategies and the alcohol exposure material. Exposure to alcohol was simulated by watching one of eight alcohol videos on the mobile phone (eg, beer, red or white wine, mixed alcoholic drinks, hard liquor), which allowed individuals to select their preferred beverage as the exposure material. The alcohol exposure videos imitated sessions with a therapist, and the alcohol presented in the videos became increasingly appetitive during the exposure session to induce cue-controlled cravings. The app contained a direct phone number to a CET therapist in case of uncontrollable cravings, and the app was only accessible during the opening hours of the alcohol outpatient clinic (Monday to Friday from 9 am to 6 pm) so that the patient could meet the therapist and get help to control the cravings. Individuals could practice exposure once a day, four times a week (a maximum of 32 sessions of approximately 15 minutes each), and they were supposed to receive a reminder every week. Due to technical issues, the software failed to send text messages to remind patients to use the app on a regular basis.

#### Aftercare as Usual

Aftercare as usual consisted of one individual follow-up session during which individuals were asked how they were doing and, if needed, were offered a brush-up of the coping strategies taught during primary treatment with cognitive behavioral therapy. The aftercare as usual session was offered 8 weeks after discharge from primary treatment, and the session lasted 60 minutes.

### Measures

#### Primary Treatment Measures

Demographic data were recorded for all patients when they entered primary alcohol treatment. The AUD assessment using the *International Classification of Diseases, Tenth Revision* Diagnostic Criteria for Research [37,38]) and the Addiction Severity Index [39,40] formed part of the routine clinical investigation and was carried out for all individuals at both the start and completion of the primary treatment. The Addiction Severity Index measured domains related to addictive behavior, including an alcohol domain indicating the severity and remission state of the AUD [39,41].

#### Cue Exposure Therapy Aftercare Outcome Measures

After primary treatment and before randomization, individuals fulfilling the eligibility criteria were further assessed on measures aimed at assessing primary and secondary outcomes to determine the effects of CET aftercare treatment. Data were collected at preaftercare (ie, after primary treatment but before entering aftercare), after 2 months (at postaftercare treatment), and again at 6 months (completion of aftercare treatment).

##### Primary Study Outcomes: Alcohol Consumption

Alcohol consumption measures were derived from Timeline Follow Back, which involves using a calendar to identify alcohol consumption patterns in the last 30 days [42-44]. The following variables were derived from this measure: sensible drinking (drinking ≤14 drinks per week for women and ≤21 drinks per week for men, as well as ≤5 drinks per day for both genders as recommended by the Danish Health Authorities [45]), abstinence, drinking days, and days with excessive drinking (drinking >5 drinks per day, where one drink contains 12 grams of ethanol) in the past 30 days.

##### Secondary Study Outcomes: Cravings and Urge-Specific Coping Skills

The Visual Analog Scale (VAS) comprises single items used to measure the degree of alcohol cravings on scales ranging from 0 to 10, with 0 representing no cravings and 10 representing extreme cravings. The scale is presented visually on a ruler; individuals were requested to report the mean level and the peak level of cravings experienced during the last 30 days [46-48].

The 22-item Urge-Specific Coping Skills Questionnaire (USCSQ) assesses 11 coping strategies that the patient may currently be using and the effectiveness of each of these when experiencing cravings or urges to drink. The strategies include those taught in primary cognitive behavioral therapy treatment and/or the CET intervention groups. Items are rated on scales ranging from 0 (never) to 10 (always) [7].

Although the routine assessments were conducted by the outpatient clinic staff, the primary and secondary study outcomes were assessed by research assistants. Attempts were made to retain participants in posttreatment assessments, even if they withdrew from aftercare.

##### Real-Time Measures of Cue-Induced Cravings

Participants in the two experimental CET arms were also asked to rate the degree of real-time cue-induced cravings experienced. Urges were measured on scales ranging from 0 (no urges) to 10 (severe urges) at three different time points: (1) at baseline (before exposure), (2) when the urge was expected to peak (during exposure), and (3) at the endpoint (after exposure) [7]. These real-time cue-induced urges were assessed by therapists for CET group and by software for CET app.

### Data Analysis

Sample characteristics were described for sociodemographic, primary, and secondary measures at baseline using frequencies for categorical variables, mean and standard deviation (SD) for normally distributed variables, and median and interquartile range (IQR) for nonnormally distributed variables.

#### Real-Time Cue-Induced Cravings

The real-time cue-induced craving measures were analyzed using Wilcoxon rank test (Mann-Whitney) to determine whether the experimental groups differed from each other. Proxy measures were calculated for the intensity of the urge induced by the selected exposure video and the effectiveness of the selected USCS in reducing the urge. The first measure was calculated by subtracting the baseline measure from the peak measure. The effectiveness of the USCS was calculated by subtracting the endpoint measure from the peak measure.

#### Aftercare Treatment Outcomes

Generalized linear mixed models were used to examine the trajectories of the primary and secondary outcomes by group allocation over time. Specifically, we wanted to examine how alcohol consumption, cravings, and USCS were influenced by the variable time point (preaftercare, postaftercare, and 6-month follow-up) and the interaction between time point and group (CET group, CET app, and aftercare as usual).

The alcohol consumption outcomes for sensible drinking and abstinence were coded dichotomously and analyzed with mixed-effects logistic regression models. Drinking days and days with excessive drinking met the assumptions for mixed-effects linear regression models and were analyzed accordingly. Similarly, cravings and USCS were analyzed with mixed-effects linear regression models.

Fixed effects consisted of time point and group×time point interaction. Because patients were randomized into three groups, this interaction tests for the existence of a treatment effect over time. All models included a subject-specific random intercept and allowed for a subject-specific random slope over time. Assuming the dropout mechanism is missing at random, these models deal efficiently with missing values due to dropout using the maximum likelihood estimator (missing were 7%, n=11 in both the first and second follow-ups). Therefore, with the mixed-effects model approach, all available data were used. If there was an overall significant interaction effect between group and any one time point, we examined whether the change over time differed between the groups and the time point using contrasts.

Two analyses were performed for each outcome measure: (1) intention-to-treat analysis (ie, irrespective of whether individuals had completed the interventions or were reinterviewed), and (2) completer (on-treatment) analyses for AUD individuals who had completed the respective intervention (completed all four CET with USCS sessions at least once or completed the aftercare as usual). The significance level was set at 5%, and two-sided analyses were conducted. All analyses were conducted using Stata version 15.

#### Sample Size

The sample size was calculated based on sensible drinking, which represents abstinence and not drinking excessively at any occasion or over time (ie, ≤5 standard drinks on one occasion, or drinking ≤14 drinks for women or ≤21 drinks for men per week). The power calculation was estimated from quality assurance and research data from the outpatient alcohol treatment clinic participating in this study. With the current treatment regimen, 65% of the patients have sensible drinking habits 6 months after treatment (information stemming from local continuous monitoring of the treatment quality). To detect an effect by comparing the three groups, a sample of 100 patients in each group was needed for 90% power of detecting a difference corresponding to an improvement of 18% using a 5% level of statistical significance.

### Statement of Ethics

The study protocol was approved by the Regional Scientific Ethical Committees for Southern Denmark (Project-ID S-20140176) and was conducted according to the World Medical Association Declaration of Helsinki.

## Results

### Sample Characteristics

During the inclusion period, 323 individuals with AUD who fulfilled the eligibility criteria concluded primary treatment and were offered participation in the aftercare treatment study. Of these, 159 individuals declined to participate, and 164 (51%) were enrolled in the study and completed preaftercare assessment (see Multimedia Appendix 1). A total of 153 of 164 (93%) individuals completed the postaftercare assessment and the 6-month follow-up: 94% (51/54) in CET group, 91% (49/54) in CET app, and 95% (53/56) in aftercare as usual.

As shown in Table 1, approximately 70% of the sample was relatively well-educated having completed either vocational training, a bachelor degree at vocational academies or university colleges (≤4 years), or a university degree or other higher education (>4 years) after finishing elementary school or high school. Approximately 50% of the sample were employed, and 10% were students receiving grants, state loans, and employment income. Approximately 35% were pensioned (mainly due to retirement), and the rest of the sample was temporarily out of employment, on sickness benefit, unemployment benefit, or cash assistance.

The preaftercare alcohol consumption measures indicated that the sample was successfully treated during the primary treatment course: 80% (132/164) achieved sensible drinking and 70% (115/164) achieved total abstinence. Among individuals who reported being nonabstinent (n=46), the median number of drinking days was 3 (IQR 4), and days with excessive drinking was median 1.5 (IQR 2). In addition, the level of cravings ranged from low to moderate on the VAS. Before initiation of the aftercare interventions, the use and perceived effectiveness of the USCS were high, particularly for “thinking about the negative consequences of drinking” and “thinking about the positive consequences of sobriety.” This could be expected given that the primary psychological treatment was cognitive behavioral therapy.

### Aftercare Treatment Retention

During the aftercare intervention, 81% (44/54) of the individuals attended at least one CET group session, 78% (42/54) attended two CET group sessions, 74% (40/54) attended three CET group sessions, and 67% (36/54) participated in all four group sessions. Similarly, 78% (42/54) completed at least one CET app session, 72% (39/54) completed two CET app sessions, 53% (29/54) completed CET app sessions, and 41% (22/54) completed at least four CET app sessions. In total, 73% (41/56) completed aftercare as usual.

#### Real-Time Cue-Induced Cravings

In line with the low level of cravings as measured by the VAS, a low intensity of real-time cue-induced cravings was observed during CET with USCS treatment. From the beginning of the alcohol exposure session until the urge intensity was supposed to peak, the CET group reported a median increase of 2.25 (IQR 2.46); when applying the USCS from the peak urge until the session ended, the median reduction was 2.25 (IQR 2.88). The CET app group showed a median increase of 0.19 (IQR 2.08) from the onset of exposure to the peak measure, followed by a median reduction of 0.37 (IQR 2.0).

### Aftercare Treatment Outcomes

As shown in Table 2, there were significant effects of time on all primary outcomes. Although the decrease in predicted mean sensible drinking from preaftercare to postaftercare was not significant (predicted mean change [PMC] −0.06, SE 0.04, *z*=−1.62, *P*=.11), the decrease in sensible drinking from preaftercare to 6-month follow-up was significant (PMC −0.17, SE 0.05, *z*=−3.07, *P*=.002) as was the decrease in abstinence from both preaftercare to postaftercare (PMC −0.12, SE 0.04, *z*=−3.15, *P*=.002) and to 6-month follow-up (PMC −0.27, SE 0.06, *z*=−4.92, *P*<.001). In line with these findings, fwe found significant increases in drinking days from preaftercare to postaftercare (PMC 1.18, SE 0.43, *z*=2.74, *P*=.006) and to 6-month follow-up (PMC 3.08, SE 0.61, *z*=5.03, *P*<.001), as well as in days with excessive drinking from preaftercare to postaftercare (PMC 1.03, SE 0.33, *z*=3.08, *P*=.002) and to 6-month follow-up (PM change 2.13, SE 0.46, *z*=4.60, *P*<.001).

Taken together, these changes showed that the total sample increased their drinking following treatment conclusion and following aftercare, and even began to relapse.

**Figure 2:**
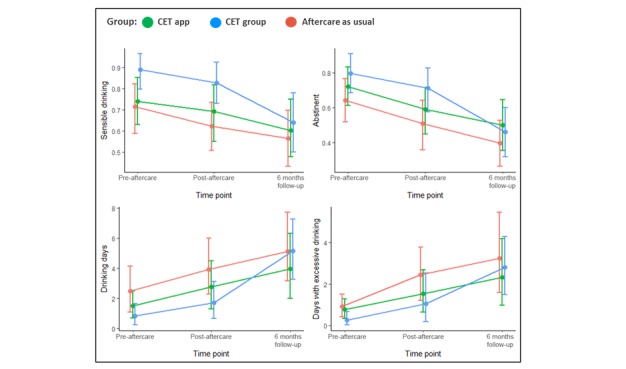
Primary outcomes by group allocation over time among individuals receiving cue exposure therapy (CET) as group aftercare or as a mobile phone app, or aftercare as usual.

**Figure 3:**
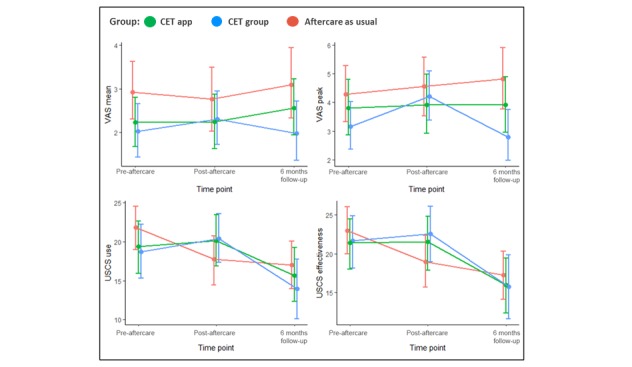
Secondary outcomes by group allocation over time among individuals receiving cue exposure therapy (CET) as group aftercare or as a mobile phone app, or aftercare as usual. USCS: urge-specific coping skills (a lower score reflects less use or lower perceived effectiveness); VAS: visual analog scale (a lower score reflects fewer alcohol cravings).

Table 3 and Figures 2 and 3 illustrate the intention-to-treat analyses for the trajectories of primary and secondary outcomes by group allocation over time.

**Table 1:** Sample characteristics before aftercare treatment with cue exposure treatment (CET) or aftercare as usual (N=164).

Sample characteristics			CET app (n=54)	CET group (n=54)	Aftercare as usual (n=56)
**Demographics**					
	Age (years), mean (SD)		46 (14)	48 (13)	45 (12)
	Age (years), range		19-68	18-80	23-69
	Male, n (%)		39 (72)	45 (83)	43 (77)
	**Further education, n (%)**				
		None	16 (30)	11 (21)	10 (19)
		≤4 years	34 (63)	37 (71)	39 (74)
		>4 years	4 (7)	4 (8)	4 (8)
	**Income, n (%)**				
		Employed	29 (54)	24 (44)	27 (48)
		Temporarily unemployed	12 (22)	10 (19)	18 (32)
		Student	3 (6)	6 (10)	5 (9)
		Pensioned	12 (22)	16 (30)	5 (9)
	Disulfiram, n (%)		39 (21)	28 (15)	34 (19)
**Alcohol consumption**					
	Sensible drinking, n (%)		40 (74)	48 (89)	40 (71)
	Abstinence, n (%)		39 (72)	46 (80)	36 (64)
	Drinking days, median (IQR^a^)		0 (2)	0 (0)	0 (2)
	Days with excessive drinking, median (IQR)		0 (1)	0 (0)	0 (1)
**Alcohol cravings (Visual Analog Scale), median (IQR)**					
	Highest urge		3 (7)	2 (6)	4 (8)
	Mean urge		2 (3)	1 (4)	3 (5)
**Urge-specific coping skills (USCSQ^b^), median (IQR)**					
	**Use**				
		Waiting it out	3 (8)	4 (9)	5 (9)
		Thinking about negative consequences	5 (8)	7 (7)	8 (6)
		Thinking about the positive consequences	8 (8)	8 (8)	8 (5)
		Alternative food and beverage intake	0 (0)	0 (0)	0 (1)
	**Effectiveness**				
		Waiting it out	5 (10)	4.5 (9)	5 (9)
		Thinking about the negative consequences	8 (8)	8 (5)	8 (6)
		Thinking about the positive consequences	9 (5)	8 (5)	8 (5)
		Alternative food and beverage intake	0 (0)	0 (0)	0 (1)

^a^IQR: interquartile range.

^b^USCSQ: urge-specific copings skills questionnaire.

**Table 2:** Predicted means of primary and secondary outcomes over time.

Time point			Overall, PM^a^ (SE)	Overall change from pretreatment, PMC^b^ (SE)	Time	
					*z*	*P* value
**Primary outcomes**						
	**Sensible drinking**					
		Pretreatment	0.77 (0.03)	—^c^	—	—
		Posttreatment	0.71 (0.04)	−0.06 (0.04)	−1.62	.11
		6-month follow-up	0.61 (0.06)	−0.17 (0.05)	−3.07	.002
	**Abstinence**					
		Pretreatment	0.71 (0.03)	—	—	—
		Posttreatment	0.60 (0.05)	−0.12 (0.04)	−3.15	.002
		6-month follow-up	0.44 (0.06)	0.27 (0.06)	−4.92	<.001
	**Drinking days**					
		Pretreatment	1.65 (0.34)	—	—	—
		Posttreatment	2.83 (0.46)	1.18 (0.43)	2.74	.006
		6-month follow-up	4.73 (0.63)	3.08 (0.61)	5.03	<.001
	**Days with excessive drinking**					
		Pretreatment	0.66 (0.13)	—	—	—
		Posttreatment	1.69 (0.35)	1.03 (0.33)	3.08	.002
		6-month follow-up	2.80 (0.48)	2.13 (0.46)	4.60	<.001
**Secondary outcomes**						
	**VAS^d^ mean**					
		Pretreatment	2.40 (0.18)	—	—	—
		Posttreatment	2.45 (0.20)	0.05 (0.20)	0.23	.82
		6-month follow-up	2.56 (0.21)	0.15 (0.22)	0.68	.50
	**VAS peak**					
		Pretreatment	3.75 (0.27)	—	—	—
		Posttreatment	4.23 (0.28)	0.48 (0.26)	1.88	.06
		6-month follow-up	3.85 (0.28)	0.10 (0.29)	0.33	.74
	**USCS^e^ use**					
		Pretreatment	20.02 (0.93)	—	—	—
		Posttreatment	19.47 (0.95)	−0.55 (1.01)	−0.54	.59
		6-month follow-up	15.66 (1.02)	−4.36 (1.02)	−4.27	<.001
	**USCS effectiveness**					
		Pretreatment	22.04 (0.95)	—	—	—
		Posttreatment	21.05 (1.03)	−0.99 (1.10)	−0.90	.37
		6-month follow-up	16.41 (1.06)	−5.63 (1.22)	−4.60	<.001

^a^PM: predicted mean.

^b^PMC: predicted mean change.

^c^Not applicable.

^d^VAS: Visual Analog Scale (alcohol cravings).

^e^USCS: urge-specific coping skills.

**Table 3:** Predicted mean differences of primary and secondary outcomes over time and by group allocation.

Time point			CET^a^ app/AAU^b^, PMD^c^ (SE)	Time × group		CET group/AAU, PMD (SE)	Time × group		CET app/CET group, PMD (SE)	Time × group	
				*z*	*P* value		*z*	*P* value		*z*	*P* value
**Primary outcomes**											
	**Sensible drinking**										
		Posttreatment	0.06 (0.09)	0.63	.53	0.03 (0.10)	0.31	.76	0.02 (0.09)	0.25	.78
		6-month follow-up	0.02 (0.12)	0.17	.86	−0.07 (0.13)	−0.57	.57	0.09 (0.12)	0.78	.44
	**Abstinence**										
		Posttreatment	0.04 (0.08)	0.45	.65	0.08 (0.09)	0.97	.33	−0.05 (0.08)	−0.58	.56
		6-month follow-up	0.07 (0.10)	0.69	.49	−0.01 (0.09)	−0.11	.91	0.08 (0.11)	0.69	.49
	**Drinking days**										
		Posttreatment	−0.23 (1.10)	−0.21	.83	−0.53 (1.02)	−0.52	.61	0.30 (1.05)	0.28	.78
		6-month follow-up	−0.30 (1.56)	−0.19	.85	1.61 (1.44)	1.12	.26	−1.91 (1.50)	−1.27	.20
	**Days with excessive drinking**										
		Posttreatment	−0.82 (0.76)	−1.08	.28	−0.78 (0.89)	−0.87	.39	−0.05 (0.78)	−0.06	.95
		6-month follow-up	−0.89 (1.19)	−0.74	.46	0.13 (1.12)	0.12	.91	−1.02 (1.07)	−0.95	.34
**Secondary outcomes**											
	**VAS^d^ mean**										
		Posttreatment	0.19 (0.51)	0.37	.71	0.45 (0.47)	0.95	.34	−0.26 (0.50)	−0.52	.60
		6-month follow-up	0.19 (0.58)	0.33	.74	−0.23 (0.52)	−0.44	.66	0.42 (0.56)	0.75	.45
	**VAS peak**										
		Posttreatment	−0.18 (0.69)	−0.26	.80	0.77 (0.58)	1.34	.18	−0.94 (0.62)	−1.54	.12
		6-month follow-up	−0.38 (0.74)	−0.51	.61	−0.93 (0.70)	−1.32	.19	0.55 (0.72)	0.76	.44
	**USCS^e^ use**										
		Posttreatment	4.90 (2.26)	2.17	.03	5.99 (2.59)	2.31	.02	−1.09 (2.52)	−0.43	.67
		6-month follow-up	1.22 (2.29)	0.53	.60	0.29 (2.65)	0.11	.91	0.93 (2.54)	0.37	.71
	**USCS effectiveness**										
		Posttreatment	4.35 (2.48)	1.75	.08	5.05 (2.93)	1.72	.09	−0.70 (2.65)	−0.26	.79
		6-month follow-up	0.47 (2.76)	0.17	.86	−0.06 (3.16)	−0.02	.99	0.53 (3.03)	0.18	.86

^a^CET: cue exposure therapy.

^b^AAU: aftercare as usual.

^c^PMD: predicted mean difference.

^d^VAS: Visual Analog Scale (alcohol cravings).

^e^USCS: urge-specific coping skills.

For the primary outcomes, Table 3 and Figure 2 show that the trajectories and the intention-to-treat analysis with generalized linear mixed models detected no interactions between time point and group. That is, there were no predicted mean differences either when the two experimental CET groups were compared with aftercare as usual or when CET app was compared with CET group over time.

For the secondary outcomes, Table 3 and Figure 3 show that no interactions were detected on alcohol cravings (mean urge and highest urge) between the groups over time. However, an interaction was detected on USCS revealing that both CET app (PMD 4.90, SE 2.26, *z*=2.17, *P*=.03) and CET group (PMD 5.99, SE 2.59, *z*=2.31, *P*=.02) applied the coping strategies more than the active controls at postaftercare, but this effect was lower at the 6-month follow-up.

The completer analyses only included individuals who completed CET with USCS (ie, completed all four sessions at least once) or completed aftercare as usual. This revealed the same pattern as the intention-to-treat analysis where a main effect for time was detected in the predicted mean of all primary outcomes, but no interactions were found between group and time points. This indicated that the entire sample increased their alcohol consumption after ending primary treatment. Similarly, no significant interactions were detected on cravings measures, and both CET app (PMD 8.35, SE 3.14, *z*=2.66, *P*=.008) and CET group (PMD 8.53, SE 3.20, *z*=2.66, *P*=.008) used the USCS more than aftercare as usual. The completer analysis also showed that the experimental groups found the USCS more effective than did the active controls (CET app: PMD 8.46, SE 3.01, *z*=2.74, *P*=0.006; CET group: PMD 7.73, SE 3.51, *z*=2.26, *P*=.02).

## Discussion

### Principal Findings

The objectives of this study were to investigate whether CET based on a treatment manual and combined with USCS delivered as alcohol treatment aftercare would increase the effectiveness of cognitive behavioral therapy, and whether delivery by means of a mobile phone app would demonstrate similar effectiveness as group sessions. Contrary to our a priori hypothesis, we did not find support for superior effectiveness of CET compared with aftercare as usual on alcohol consumption and urge-related outcomes over time. The experimental CET groups used USCS more than the controls at posttreatment, but this effect was reduced at 6-month follow-up. We found no differences in outcomes between the experimental groups receiving CET through group sessions or a mobile phone app.

Our findings are similar to the previous meta-analysis indicating that individuals with AUD exposed to CET showed no effect to small overall additional effects on drinking-related outcomes [6]. However, the meta-analysis also suggested that CET combined with USCS may achieve more favorable outcomes than conventional CET [28,29]. Monti et al [28] investigated the effectiveness of CET with USCS compared with relaxation and meditation therapy for increasing abstinence among 40 inpatients with AUD on otherwise standard treatment. The 22 individuals who received CET achieved superior results on several alcohol consumption outcomes, including abstinence, fewer drinking days, and drinks per drinking day at 6-month follow-up [28]. Rohsenow et al [29] conducted a larger RCT with 100 individuals with AUD, of whom 59 received CET with USCS as an add-on to standard inpatient treatment. Again, the active control condition was relaxation and meditation therapy, and the treatment goal was abstinence. Patients receiving CET with USCS reported fewer days with excessive drinking at 6- and 12-month follow-ups [29]. In further support of these findings, Monti et al [30] reported similar positive drinking outcomes among inpatients at 6- and 12-month follow-ups when the intervention was delivered as aftercare combined with other psychological and urge-reducing pharmacotherapy treatment. Similar to our findings, these previous studies did not find differences between the experimental and nonexperimental groups on completion of treatment and at 3-month follow-up. However, their findings at 6 and 12 months are in sharp contrast to our results.

It has been demonstrated that CET achieves more favorable outcomes when compared with active control conditions (eg, relaxation and meditation therapy) other than cognitive behavioral therapy [6]. Of four controlled trials (using either randomized or sequential allocation) comparing conventional CET to cognitive behavioral therapy, three reported an equal effect of CET on alcohol consumption outcomes [49-51], and the fourth study reported a superior effect for CET [52]. Furthermore, Kavanagh and coworkers [53] performed an RCT on individuals with AUD allocated to receive either cognitive behavioral therapy alone or cognitive behavioral therapy with CET as an add-on intervention. Results indicated that the addition of conventional CET to cognitive behavioral therapy did not improve alcohol outcomes at posttreatment or at 6- and 12-month follow-ups [53]. Although conventional CET has been compared to cognitive behavioral therapy in prior studies [49-52], and CET has been implemented as an add-on intervention to cognitive behavioral therapy [53], no studies have compared CET combined with USCS to cognitive behavioral therapy. Individuals having CET with USCS can practice coping skills while they are exposed to alcohol cues in vivo, which contrasts with most cognitive behavioral therapy approaches. However, the results from this study suggest that when CET plus USCS is added to cognitive behavioral therapy, it does not enhance the effectiveness of cognitive behavioral therapy in preventing relapses in outpatients on completion of treatment. The combination of CET with coping skills thus appears to be a less important feature for the effectiveness at 6-month follow-up.

Cue exposure therapy with or without USCS is assumed to work by reducing cue-induced cravings (eg, [1-5]). Although prior studies combining CET with USCS have found positive alcohol consumption outcomes at 6- and 12-month follow-ups, it has proven difficult to demonstrate a decrease in the degree of cravings [28,29]. Further, very few of the previous studies investigating a conventional CET approach applied psychometric outcome measures of cravings, or they only applied them at pretreatment to assess whether they predicted alcohol consumption outcomes at later follow-ups [50,52,54,55]. Our sample can generally be defined as non- or low-urge reactors, which is evident from the low craving level reported, and the experimental groups did not achieve a higher degree of cue-reactivity reduction than the control group. This is consistent with prior findings and with the notion that a clinically relevant degree of cravings is required at pretreatment; otherwise, cravings cannot be expected to decrease and may obscure the probability of detecting a change [56]. However, this does not fully explain why this RCT and prior studies did not find more significant results. The fact that it is difficult to document a decrease in cravings relative to alcohol consumption challenges the theoretical assumption that cue-induced cravings cause addictive behavior or, perhaps more likely, the way we currently measure cravings in clinical studies. Cue-induced cravings most often represent automatic and implicit cognitive processes [57,58], and current self-report craving measures may be insufficient to capture such unconscious processes unless cravings reach a certain threshold and are experienced consciously.

It was found that CET delivered through group sessions resulted in greater use of USCS compared with the control group, as reported previously [28,29]. However, this was only the case at posttreatment, and the effect attenuated at 6-month follow-up, suggesting that the advantages may be short-lived. Further, as our overall sample reported low levels of alcohol cravings, they may not have used the USCS or perceived it to be ineffective in reducing the cravings.

The results indicate that the mobile phone app featuring CET with USCS may be equivalent in effectiveness to CET delivered in group sessions for improving alcohol consumption, craving, and USCS outcomes. However, if CET through any delivery pathway does not increase the effectiveness of the already well-documented approach of cognitive behavioral therapy, there is no need to implement CET as an add-on or aftercare. Indeed, this is the first study to show that CET with USCS may not be an effective psychological approach. Prior studies did not implement the experimental approaches as aftercare or in extension to primary treatment with cognitive behavioral therapy, but instead as add-on to primary treatment with other interventions (eg, community meetings, alcohol and health education, vocational counseling, 12-step meetings). It is plausible that we found no effect of CET due to the short follow-up period. The effect of aftercare may require longer than 6 months, and more so when primary treatment results in such a well-treated sample as that in this study, with almost no alcohol intake or cravings. Prior studies have applied CET with USCS among inpatients with a higher degree of addiction severity and using abstinence as the only treatment goal, suggesting that the approach may be more effective in specific patient populations. More research is thus warranted to determine whether CET with USCS is an effective approach. If it does prove effective under certain conditions, we have shown that the method can easily be implemented into a mobile phone app with apparently the same effect as group sessions. In addition to reducing treatment costs, this may have other advantages such as greater access to psychological treatment for treatment-seeking and non-treatment-seeking AUD populations.

### Strengths and Limitations

This study is the largest RCT conducted in the area and was based on the CONSORT statement for RCT studies, which was not customary in previous studies. Further strengths are that we eliminated selection bias at entry to aftercare treatment by conducting the study as an RCT, and we based the experimental interventions on a published treatment manual, which optimized the replicability of the study and future implementation of the app as an evidence-based treatment. A high postaftercare follow-up rate of 93% was achieved, which heightens the power and generalizability of the study and reduces the risk of bias. Finally, the findings from this RCT can guide the development of evidence-based eHealth interventions, which is important given the current widespread availability of questionable AUD treatments in app stores.

A number of limitations should also be mentioned. Although the RCT is the largest conducted to date, it may still lack power to detect potential effects and hence commit type II errors, particularly in view of the well-treated sample. Indeed, the power calculation led us to aim for 300 participants, which could not be achieved due to surprisingly few patients entering primary treatment during the recruitment period and many patients declining to participate in the aftercare study. Second, it was challenging to get the individuals with AUD to use the app, and technical issues meant that the software failed to send regular text messages to remind patients to use the app. These limitations could be easily overcome in the future. Finally, no objective measures of alcohol consumption and cravings were applied to validate self-reported outcomes.

### Conclusion

Cue exposure therapy combined with urge-specific coping skills delivered as aftercare either in a group session or by a mobile phone app did not increase the effectiveness of cognitive behavioral therapy in this study. This is the first study to show that CET with USCS may not be an effective psychological approach for aftercare of individuals treated for AUD.

### Future Directions

There is a need for more large, high-quality RCT studies to assess the effects of CET combined with USCS for individuals with AUD. Further research is especially warranted to investigate (1) appropriate measures of alcohol consumption and craving, including objective measures; (2) the effectiveness when targeting different urge-reactor profiles; (3) the long-term effectiveness of CET, especially when delivered through a mobile device; and (4) whether the app (and also CET in general) is better suited to treat subsamples of individuals with AUD (eg, severe AUD, urge reactors, younger individuals, or those with higher education, living in rural areas, or with busy life schedules).

## Appendix

Multimedia Appendix 1 CONSORT‐EHEALTH checklist (V 1.6.1).

## Abbreviations

AUD: alcohol use disorder
CET: cue exposure therapy
CONSORT: Consolidated Standards of Reporting Trials
IQR: interquartile range
PM: predicted mean
PMC: predicted mean change
PMD: predicted mean difference
RCT: randomized controlled trial
USCS: urge-specific coping skills
VAS: Visual Analog Scale
